# Supplementary material for: Causal Relationship between Aging and Anorexia Nervosa: A White-Matter-Microstructure-Mediated Mendelian Randomization Analysis
Source: Biomedicines. 2024 Aug 16;12(8):1874. doi: 10.3390/biomedicines12081874 (PMC11351342; doi:10.3390/biomedicines12081874)
Supplement: Supplementary file 1 [file biomedicines-12-01874-s001.zip › Supplementary Figure/Figure S8/Leaveoneout plot of MR analysis for TL to ubm-b-1795.pdf]

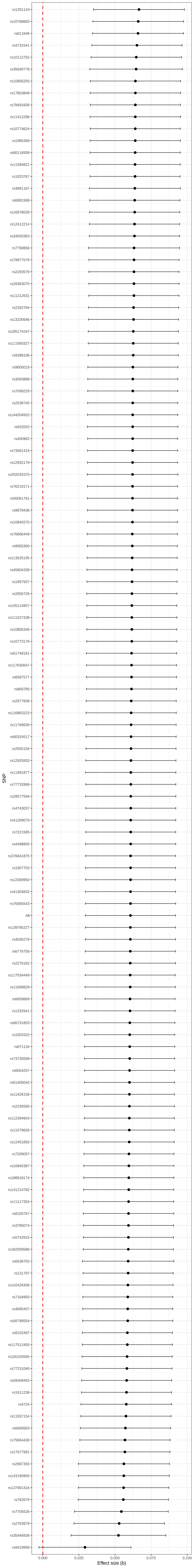
rs1291143
rs10768683
rs611646
rs4731541
rs10112752
rs35640778
rs10905255
rs17803849
rs78491606
rs11412296
rs10774624
rs1985369
rs80116508
rs11584821
rs1023767
rs3891167
rs6881568
rs16978028
rs12412214
rs34550383
rs7790856
rs79977579
rs2293579
rs28363070
rs11212631
rs2282764
rs13230646
rs185174247
rs111950327
rs9398196
rs9600019
rs3093888
rs7099229
rs2538745
rs144204502
rs932002
rs450962
rs73581419
rs12932179
rs202034370
rs76219171
rs56061761
rs9878436
rs10840270
rs76666449
rs9955360
rs113525195
rs45604339
rs1957937
rs2056726
rs145114957
rs111527438
rs10805346
rs10773176
rs61748181
rs117630647
rs6587577
rs869785
rs2977608
rs116863223
rs11769630
rs80324517
rs2555104
rs12925933
rs11991877
rs77732866
rs28577594
rs4743037
rs41269079
rs7221585
rs4498805
rs376641875
rs1907702
rs12369950
rs41304832
rs76065543
All
rs139795227
rs4530278
rs6776756
rs2276182
rs117034449
rs11699829
rs6659669
rs1332941
rs66731853
rs1003322
rs871134
rs73730598
rs6054257
rs61405042
rs11426156
rs2230590
rs112394943
rs11579626
rs12451892
rs7209057
rs10845387
rs188918174
rs141214782
rs11117354
rs8105767
rs3785074
rs5742915
rs182059586
rs6536702
rs131797
rs142426306
rs7164950
rs4695407
rs56799554
rs8102497
rs117512405
rs150150565
rs77231040
rs59409453
rs1611236
rs4724
rs11557154
rs6669563
rs75664430
rs17677991
rs2967355
rs143190905
rs137901416
rs762679
rs7705526
rs2763979
rs35446936
rs9419958
SNP
0.000
0.025
0.050
0.075
0.100
Effect size (b)
